# Anticoagulant Use in COVID-19 Patients: A Longitudinal Study From Zanjan, Iran

**DOI:** 10.7759/cureus.66798

**Published:** 2024-08-13

**Authors:** Vanoushe Azimi Pirsaraei, Manizhe Jozpanahi, Koorosh Kamali, Leila Hamzeloo, Seyedeh Pegah Saeid

**Affiliations:** 1 Research Committee/General Medicine, Zanjan University of Medical Sciences, Zanjan, IRN; 2 Department of Infectious Diseases, Valiasr Hospital, Zanjan University of Medical Sciences, Zanjan, IRN; 3 Department of Public Health, School of Public Health, Zanjan University of Medical Sciences, Zanjan, IRN

**Keywords:** covid-19, pandemic, severe acute respiratory syndrome, anticoagulants, coronavirus disease 2019

## Abstract

Background

The mortality and morbidity of thrombotic events in patients with coronavirus disease 2019 (COVID-19) are increasing worldwide. The clinical impact of prophylactic anticoagulation regimens among these patients in Iran remains unclear. This study aimed to evaluate the use of prophylactic anticoagulants and outcomes among COVID-19 patients admitted to a tertiary referral hospital.

Methodology

Patients diagnosed with COVID-19 and hospitalized between March 20 and June 20, 2020, were included in this longitudinal study after obtaining informed consent. Demographic and clinical data were collected from the hospital information system and medical records. Outcomes during this period were also evaluated. The data were entered into the preparation checklist and analyzed using SPSS version 24 software (IBM Corp., Armonk, NY, USA), employing chi-square, Fisher’s exact, and Mann-Whitney U tests.

Results

Of the 831 enrolled patients, 51.9% were female, and 10.6% needed to be admitted to the intensive care unit (ICU). The mean age of the patients was 57.16 ± 17.32 years, and the mortality rate was estimated to be 9.4%. Mortality rates were significantly higher at older ages, in men, patients with ICU admission, severe pulmonary involvement, malignancy, airway obstruction, ischemic heart disease, and previous cerebrovascular accidents. ICU admission and mortality were statistically significantly higher in those who received concurrent prophylactic anticoagulants and aspirin than in other individuals.

Conclusions

Our study demonstrated that administering prophylactic aspirin with or without anticoagulant agents in COVID-19 patients did not reduce mortality rates or ICU transfers. However, it is worth noting that anticoagulant prescription was associated with a decrease in ICU admissions, which could potentially alleviate the significantly higher mortality rates observed among ICU patients in this study. Further research is needed to explore the potential benefits of anticoagulants in COVID-19 treatment.

## Introduction

Coronavirus disease 2019 (COVID-19) is caused by the severe acute respiratory syndrome coronavirus 2 (SARS-CoV-2) which was recognized by the World Health Organization as a pandemic and a global health problem. Various manifestations in COVID-19 patients have been reported, from mild to severe symptoms, including fever, cough, shortness of breath, myalgia, systemic inflammatory response syndrome (SIRS), acute respiratory distress syndrome (ARDS), organ failure, and shock [1-3]. Elevated D-dimer level was the most common hemostatic disorder associated with COVID-19, which was linked to thrombosis, the increased need for mechanical ventilation, intensive care unit (ICU) admission, and mortality [2]. Recent studies have demonstrated that the mortality rate in patients with D-dimer levels above 1,000 ng/mL was about 20 times higher than in patients with lower D-dimer levels [4,5]. The incidence of SIRS, severe disease, decreased mobility, malignancy, previous history of venous thromboembolism (VTE), age over 70 years, and underlying risk factors predispose the patient to thrombotic events [6,7].

Thrombotic problems, particularly pulmonary thromboembolism (PTE), were more common among patients with ARDS due to COVID-19, and despite receiving anticoagulants, most patients developed life-threatening coagulation complications [8]. Despite prophylactic administration of appropriate doses of low-molecular-weight heparin, VTE in patients admitted with COVID-19 has been reported to be between 0% and 8%. In comparison, it has been reported to be between 16% and 35% among patients admitted to the ICU. In autopsies of COVID-19 patients not suspected of VTE before death, the rate of detected thrombosis was up to 58% [3].

Heparin is an effective anti-inflammatory agent and can reduce pulmonary edema and venous leakage. In addition, heparin protects endothelial cells and neutralizes histone toxicity [9]. Among the drugs prescribed to patients, aspirin, in addition to the anticoagulant, has antiplatelet and anti-inflammatory effects that prevent virus transcription and reduce lung damage. Early aspirin administration in patients with COVID-19 can help reduce disease severity, length of hospital stay, and cardiovascular complications [10].

Regarding the increased mortality and morbidity of thrombotic events in patients with COVID-19, the need for prophylactic therapy, and the lack of related research at the time of this study in Iran, this study aimed to evaluate the use of prophylactic anticoagulants and outcomes among patients with a definitive diagnosis of COVID-19 admitted to a tertiary referral hospital.

## Materials and methods

Selection and description of participants

This longitudinal study investigated all proven COVID-19 patients admitted to the COVID-19 wards of Valiasr Education and Treatment Center in Zanjan, Iran, between March 20, 2020, and June 20, 2020. Exclusion criteria were incomplete medical records and discharge against medical advice during treatment. According to the guidelines, the diagnosis of COVID-19 patients was confirmed with a positive result of SARS-CoV-2 on the reverse transcription polymerase chain reaction (RT-PCR) test or CT imaging manifestations of COVID-19. This study was approved by the Institutional Review Board of Zanjan University of Medical Sciences (approval number: IR.ZUMS.REC.1399.265).

The third edition of Iran’s Diagnostic Therapeutic Flowchart for COVID-19, released on March 10, 2020, by the Ministry of Health Science Board, listed treatments such as appropriate antiviral therapies (oseltamivir, hydroxychloroquine, lopinavir/ritonavir, atazanavir, and ribavirin), oxygen therapy, and supportive medical care. In particular situations and based on clinical judgment, a specialist practitioner may decide to administer corticosteroids and anticoagulants in patients with COVID-19 [11]. These protocols were implemented in our hospital for the treatment of COVID-19 patients.

Data collection

Information on the studied variables including age, gender, type of anticoagulants (enoxaparin/heparin/rivaroxaban used by the patient), the dose of anticoagulants, underlying diseases, aspirin intake, length of hospital stay, ICU admission, and the mortality rate was extracted from the medical records of hospitalized patients and recorded in the preparation checklist. Two board-certified radiologists independently assessed all CT images and were blinded to patient symptoms or outcomes. The initial chest CT was evaluated for the following characteristics: lesion distribution, interlobular septal thickening, ground-glass opacities, consolidation, crazy-paving pattern, and reverse halo sign. The chest CT severity score was determined by assessing the extent of involvement across the five lung lobes. Each lobe was visually scored on a scale of 0-5, where 0 represented no involvement, 1 indicated involvement of less than 5%, 2 denoted 5-25% involvement, 3 signified 26-49% involvement, 4 represented 50-75% involvement, and 5 indicated more than 75% involvement. The overall CT score was calculated by summing the scores of the individual lobes, yielding a total that ranged from 0 (indicating no involvement) to 25 (indicating maximum involvement). Using the chest CT severity score, the severity of the disease was classified as mild (score seven or less), moderate (score 7-18), and severe (score 18 or more) [12].

Statistical analysis

The samples in the study were selected using a census sampling method. After allocating appropriate codes, the data were entered into SPSS version 24 software (IBM Corp., Armonk, NY, USA) and analyzed. Descriptive statistics, including mean and standard deviation, were used for continuous quantitative variables. In addition, percentages and frequency were reported in tables for qualitative and nominal variables. Parametric or non-parametric tests (t-test/Mann-Whitney U test) were used to compare quantitative variables depending on their distribution. A chi-square test was used to assess qualitative variables. P-values <0.05 were considered statistically significant with a 95% confidence interval.

## Results

In this study, 400 (48.1%) participants were male, and 431 (51.9%) were female. Of these, 78 patients died; 46 were male (58.9%), and 32 were female (41.03%). The mortality rate of men was significantly higher (p = 0.044). The mean age of the patients at disease onset was 57.16 ± 17.32 years, with an age range of 13 to 95 years, and a median age of 58 years. Among patients, 32 (3.9%) had immunodeficiency disease, 25 (3%) had cancer, 74 (8.9%) had obstructive airway disease (OAD), 135 (16.2%) had diabetes mellitus, and 254 (30.6%) reported hypertension. Furthermore, 98 (11.8%) cases had ischemic heart disease (IHD), followed by a history of stroke (21 cases; 2.5%) and a history of PTE (3 cases; 0.4%).

Among the patients studied, 88 (10.6%) needed to be admitted to the ICU. Among 78 dead patients, 50 (64.1%) were transferred to the ICU, and 28 (35.9%) were not. Mortality was significantly higher among the patients who were transferred to the ICU. SARS-CoV-2 RT-PCR test was positive for 698 (90.2%) patients and negative for 76 (9.8%) patients. There was no statistically significant relationship between mortality and positive test results.

The mean duration of hospitalization was 7.2 ± 5.46 days, and the median was six days. The patients’ initial oxygen saturation (SpO_2_) was estimated to be 89.32 ± 7.24%, ranging between 44% and 99%. On average, 70,197.33 ± 59,508.74 units of heparin were administered to 75 patients. The mean dose of enoxaparin was 269.80 ± 233.81 mg for 349 patients, and the median dose of prednisolone for 197 patients was 100 mg.

Age-related mortality was about 9% in individuals aged <50 years, while patients aged ≥80 years showed the most remarkable fatality rate (30.76%). The reported frequency of deaths was 10.25% in 50-59-year-olds, 21.79% in 60-69-year-olds, and 28.20% in 70-79-year-olds. Table 1 shows the relationship between age, duration of hospitalization, SpO_2_, and the dose of heparin, enoxaparin, and prednisolone administration with mortality. There was a significant relationship between age, SpO_2_ at the time of admission, and the dose of prednisolone administration with mortality; hence, the mean age of the patients in the non-mortality group was higher than patients with mortality. Further, the dose of prednisolone and the percentage of blood oxygen level at the time of admission were higher in the group with mortality.

**Table 1:** Studied characteristics based on mortality. a: Mann-Whitney test. SD: standard deviation; IQR: interquartile range; SpO_2_: oxygen saturation

Characteristic		Number	Mean	SD	Median	IQR	P-value^a^
	Mortality						
Age (years)	Yes	78	71.4	24.45	81	38	<0.001
	No	753	79.25	8.86	81.5	12	
Duration of hospitalization (days)	Yes	78	23	11.74	31	20	0.748
	No	753	16.92	14.39	14	10	
Heparin (unit)	Yes	22	93,520	85,214.15	64,000	166,800	0.930
	No	53	60,416.67	58,444.15	55,000	47,500	
Enoxaparin (mg)	Yes	23	780	844.03	280	1,490	0.611
	No	326	496.67	433.91	300	660	
Prednisolone (mg)	Yes	43	652.32	704.8	226.8	1,292	0.003
	No	154	396.1	884.2	51.5	202	
Initial SpO_2_ (%)	Yes	76	88.8	5.21	90	9	<0.001
	No	746	85.25	10.92	88	9	

As shown in Table 2, the mortality rate of participants with immunodeficiency, diabetes mellitus, hypertension, and a history of PTE was not statistically significant compared to other individuals (p > 0.05). The mortality rate of patients with malignancy, OAD, IHD, and previous cerebrovascular accident (CVA) was significantly higher than that of other people (p <0.05).

**Table 2:** Frequency of mortality based on the comorbidities. Data are presented as n (%). ^a^: chi-square tests; ^b^: Fisher’s exact tests. OAD: obstructive airway disease; IHD: ischemic heart disease; CVA: cerebrovascular accident; PTE: pulmonary thromboembolism

Variables		Mortality		P-value
		Yes	No	
Immunodeficiency disease^a^	Yes	6 (18.75)	26 (81.25)	0.064
	No	72 (9.01)	727 (90.99)	
Malignancy^a^	Yes	7 (28.0)	18 (72.0)	0.001
	No	71 (8.81)	735 (91.19)	
OAD^a^	Yes	15 (20.27)	59 (79.73)	0.001
	No	63 (8.32)	694 (91.68)	
Diabetes mellitus^a^	Yes	15 (11.11)	120 (88.89)	0.453
	No	63 (9.05)	633 (90.95)	
IHD^a^	Yes	15 (15.31)	83 (84.69)	0.032
	No	63 (8.60)	670 (91.40)	
Previous CVA^a^	Yes	10 (47.62)	11 (52.38)	<0.001
	No	68 (8.40)	742 (91.60)	
PTE^b^	Yes	0 (0)	3 (100)	1.000
	No	78 (9.42)	750 (90.58)	
Hypertension^a^	Yes	27 (10.63)	227 (89.37)	0.415
	No	51 (8.84)	526 (91.16)	

The mortality rate of patients receiving hydroxychloroquine, oseltamivir, and atazanavir was not statistically significant compared to other individuals (p > 0.05) (Table 3). The difference in mortality rates between people receiving lopinavir/ritonavir, ribavirin, intravenous immunoglobulin (IVIG), and aspirin was statistically significantly higher than other individuals (p < 0.05).

**Table 3:** Mortality rate based on medication type. Data are presented as n (%). ^a^: chi-square tests; ^b^: Fisher’s exact tests. IVIG: intravenous immunoglobulin

Variables		Mortality		P-value
		Yes	No	
Hydroxychloroquine^a^	Yes	70 (9.01)	707 (90.99)	0.157
	No	8 (14.82)	46 (85.18)	
Lopinavir/ritonavir^a^	Yes	36 (17.65)	168 (82.35)	<0.001
	No	42 (6.70)	585 (93.30)	
Oseltamivir^a^	Yes	15 (14.42)	89 (85.58)	0.060
	No	63 (8.67)	664 (91.33)	
Ribavirin^b^	Yes	4 (44.44)	5 (55.56)	0.006
	No	74 (9.00)	748 (91.00)	
Atazanavir^a^	Yes	38 (11.73)	286 (88.27)	0.064
	No	40 (7.89)	467 (92.11)	
IVIG^b^	Yes	4 (50.00)	4 (50.00)	0.004
	No	74 (8.99)	749 (91.01)	
Aspirin^a^	Yes	28 (15.22)	156 (84.78)	0.002
	No	50 (7.73)	597 (92.27)	

Of the 831 patients, 218 (28.31%) had severe involvement based on the lung CT scan, followed by 313 (40.65%) with moderate involvement, 227 (29.48%) with mild involvement, and 12 (1.56%) without involvement. Among 78 patients who died, 50 (69.45%) patients with severe involvement were reported, followed by 15 (20.83%) patients with moderate involvement, and seven (9.72%) patients with mild involvement. None of the individuals died without pulmonary involvement. The mortality rate of participants with severe pulmonary involvement was statistically significantly higher than others. No information was available on the pulmonary involvement of 61 patients.

As shown in Table 4, the rate of ICU transfer was higher in the group receiving concomitant aspirin and anticoagulant than in the group receiving neither. In addition, the ICU admission rate was statistically significantly higher in patients taking both anticoagulant and aspirin than in patients taking at least one anticoagulant.

**Table 4:** Comparison of ICU transfers based on aspirin/anticoagulant administration. Data are presented as n (%). ICU: intensive care unit

Variables	Transfer to ICU		P-value
	Yes	No	
No aspirin or anticoagulant	19 (5.25)	343 (94.75)	<0.001
Both aspirin and anticoagulant	29 (23.97)	92 (76.03)	
Low-dose aspirin only (80 mg)	8 (12.50)	56 (87.50)	0.064
Both aspirin and anticoagulant	29 (23.97)	92 (76.03)	
At least one anticoagulant	32 (11.31)	251 (88.69)	0.001
Both aspirin and anticoagulant	29 (23.97)	92 (76.03)	

The mortality rate was higher in the group receiving concomitant aspirin and anticoagulants than in the non-receiving group. Mortality was lower in the group receiving anticoagulants alone than in the group receiving concomitant aspirin and anticoagulants (Table 5).

**Table 5:** Comparison of mortality rate based on aspirin/anticoagulant administration. Data are presented as n (%).

Variables	Mortality		P-value
	Yes	No	
No aspirin or anticoagulant	27 (7.46)	335 (92.54)	0.015
Both aspirin and anticoagulant	18 (14.88)	103 (85.12)	
Low-dose aspirin only (80 mg)	11 (17.19)	53 (82.81)	0.681
Both aspirin and anticoagulant	18 (14.88)	103 (85.12)	
At least one anticoagulant	22 (7.77)	261 (92.23)	0.029
Both aspirin and anticoagulant	18 (14.88)	103 (85.12)	

## Discussion

The COVID-19 pandemic, induced by SARS-CoV-2, continues to be a global concern, and the best strategy for disease control involves taking preventative measures, identifying the infection early, and developing efficient treatment regimens [13]. The present study investigated the clinical outcomes of COVID-19 patients treated with aspirin, prophylactic anticoagulants, or both. Similar to our results in age-related mortality, Bonanad et al. (2020) showed that patients under 50 had a death rate of less than 1.1%, while patients above 80 had the most significant mortality rate. Patients between the ages of 60 and 69 years showed the highest increase in mortality compared to those between 50 and 59 years [14].

In this study, the hospital mortality rate among men and ICU patients was statistically more significant compared to women and non-ICU patients. Consistent with our results, recent studies revealed that the death rate of COVID-19 was more significant in males and ICU patients. Most critically ill patients with COVID-19 in the ICU required invasive mechanical ventilation, and the mortality rate was higher than others. Males may have a greater risk of death due to increased comorbidities and delayed viral RNA clearance in males. In general, females have more potent immune responses in comparison to males [15,16].

OAD and IHD were associated with increased mortality rates in this study. The underlying chronic obstructive pulmonary disease and IHD are significantly associated with worse prognosis in patients with COVID-19. Therefore, patients with these diseases should be identified as high-risk groups, and preventive measures and appropriate management for COVID-19 should be considered [17,18].

Overall, 777 out of 831 studied patients received hydroxychloroquine during hospitalization. There was no significant difference in mortality between drug recipients and non-recipients. In a meta-analysis, the evidence demonstrated that hydroxychloroquine, with or without azithromycin, did not affect the risk of hospital admission in COVID-19 outpatients and short-term mortality in hospitalized patients [19]. In the current investigation, the death rate among those who received lopinavir/ritonavir was statistically substantially greater than non-recipients. In a review by Joseph et al., there was no effect of lopinavir/ritonavir in treating COVID-19, improving outcomes, chest CT clearing, and death rate drop. In COVID-19 patients, adding lopinavir-ritonavir to standard therapy provides little benefit [20].

According to the results of this study, patients who received IVIG had a greater mortality rate than non-recipients. The reason might be the indication of using this drug in critically ill patients who were already at a greater risk of mortality because of the severity of their condition. In a study by Sakoulas et al., IVIG was shown to significantly improve hypoxia, reduce hospitalization time, and reduce the need for mechanical ventilation [21]. Tabarsi et al. found that using IVIG in combination with hydroxychloroquine and lopinavir/ritonavir in patients with severe illness did not benefit disease severity and outcomes [22].

In total, 184 individuals out of 831 were given low-dose aspirin throughout their hospitalization for various reasons. Patients who received the medicine died at a considerably greater rate than non-recipients. A cohort study of 21,998 patients found that the prescription of aspirin before the diagnosis of COVID-19 seemed to increase the mortality rate, and aspirin usage following the diagnosis increased the rate of conventional oxygen therapy [23].

In our study, patients who used concurrent prophylactic anticoagulants and aspirin at the same time had more significant mortality and ICU admission incidence than others. Santoro et al. enrolled 8.168 hospitalized COVID‐19 patients to evaluate the impact of aspirin therapy on prophylactic anticoagulation (PAC). The results showed that the combination of PAC and aspirin was linked with a reduced risk of death compared to PAC alone [24]. A study by Matli et al. suggested that a combination of therapeutic anticoagulation and antiplatelet therapy, or therapeutic anticoagulation alone, was related to better prognosis in COVID-19 hospitalized patients compared to patients receiving PAC as the standard of care in current guidelines. Furthermore, compared to PAC alone, PAC with antiplatelet treatment dramatically declined the need for invasive mechanical ventilation [25].

In a meta-analysis, Yasuda et al. found that prophylactic or therapeutic anticoagulants reduced mortality and VTE in individuals with moderate COVID-19 compared to no treatment. While there was no change in the incidence of mortality between the prophylactic and therapeutic groups, the incidence of VTE was considerably lower with the treatment dose [26]. Because of the disparity in the results of the studies, further research with larger sample sizes is required in Iran to obtain more reliable results.

Patients with severe lung involvement were more likely to die or require ICU admission in this study. The degree of lung involvement on the initial CT was independently linked with the outcome in the study by Ruch et al. Compared with those with less than 50% involvement, more patients with greater than 50% involvement progressed to severe disease [27].

In our investigation, patients with SpO_2_ of less than 90% had a significantly higher death and ICU admission rate. Consistent with our findings, in a retrospective cohort study, SpO_2_ of less than 90% at the diagnostic time was a substantial predictor of in-hospital death in COVID-19 patients. The risk of death was 1.86, 4.44, and 7.74 times higher for patients with SpO_2_ of 89-85%, 84-80%, and <80%, respectively, compared to those with SpO_2_ >90% [28].

Limitations

As this was a single-center, urban-based study, the data may not represent the entire population of Iran. The in-hospital outcomes of the patients were investigated, while the outcomes after discharge from the hospital were not considered. Additionally, as the study was observational, some uncertainty remained that can be resolved through randomized trials. Another limitation is that our patients were treated throughout the first year of the pandemic, a period when COVID-19 treatment strategies were significantly evolving based on emerging scientific evidence.

## Conclusions

Our study demonstrated that administering prophylactic aspirin with or without anticoagulant agents in COVID-19 patients did not reduce mortality rates or ICU transfers. However, it is worth noting that anticoagulant prescription was associated with a decrease in ICU admissions, which could potentially alleviate the significantly higher mortality rates observed among ICU patients in this study. Further research is needed to explore the potential benefits of anticoagulants in COVID-19 treatment.
